# AttBiomarker: unveiling preeclampsia biomarkers and molecular pathways through two-stage gene selection techniques and attention-based CNN with gene regulatory network analysis

**DOI:** 10.1093/bib/bbaf473

**Published:** 2025-09-16

**Authors:** Sakib Sarker, S M Hasan Mahmud, Md Faruk Hosen, Kah Ong Michael Goh, Watshara Shoombuatong

**Affiliations:** Department of Computer Science and Engineering, Uttara University, Turag, Uttara, Dhaka 1230, Bangladesh; Department of Software Engineering, Daffodil International University, Daffodil Smart City (DSC), Birulia, Savar, Dhaka 1216, Bangladesh; Centre for Advanced Machine Learning and Applications (CAMLAs), Dhaka 1229, Bangladesh; Centre for Advanced Machine Learning and Applications (CAMLAs), Dhaka 1229, Bangladesh; Department of Computing and Information System, Daffodil International University, Daffodil Smart City (DSC), Birulia, Savar, Dhaka 1216, Bangladesh; Center for Image and Vision Computing, COE for Artificial Intelligence, Faculty of Information Science & Technology (/FIST), Multimedia University, Jalan Ayer Keroh Lama, Bukit Beruang Melaka 75450, Malaysia; Center for Research Innovation and Biomedical Informatics, Faculty of Medical Technology, Mahidol University, Bangkok 10700, Thailand

**Keywords:** preeclampsia, attention-based CNN, feature selection, differential expression, hub genes, biomarkers

## Abstract

Preeclampsia is a complex pregnancy disorder that poses significant health risks to both mother and fetus. Despite its clinical importance, the underlying molecular mechanisms remain poorly understood. In this study, we developed an integrative deep learning and bioinformatics approach to identify potential biomarkers for preeclampsia. Three microarray datasets related to preeclampsia were initially analyzed to select a preliminary gene subset based on $P$-values. Feature selection was then performed in two consecutive rounds: first, the Fisher score method was applied to extract significant genes, followed by the minimum Redundancy Maximum Relevance method to refine the subset further. These selected gene subsets were trained using our proposed Attention-based Convolutional Neural Network (AttCNN), which achieved the highest classification accuracy compared with other models. From the experiments, a set of 58 common genes was identified between differentially expressed genes and the final optimized subset. Here, Gene Ontology and KEGG pathway enrichment analyses highlighted key biological processes and pathways associated with preeclampsia. Subsequently, a protein–protein interaction network was constructed, identifying 10 hub genes: TSC22D1, IRF3, MME, SRSF10, SOD1, HK2, ERO1L, SH3BP5, UBC, and ZFAND5. Further analysis of gene regulatory networks, including transcription factor–gene, gene–microRNA, and drug–gene interactions, revealed that seven hub genes (HK2, SRSF10, SOD1, ERO1L, IRF3, MME, and SH3BP5) were strongly associated with preeclampsia. Molecular docking analysis showed that HK2, SH3BP5, and SOD1 exhibited significant binding affinities with two preeclampsia drugs. These findings suggest that the identified hub genes hold promise as biomarkers for early prognosis, diagnosis, and potential therapeutic targets for preeclampsia.

## Introduction

Preeclampsia (PE) is a pregnancy-related disorder that usually develops after the 20th week of gestation. It is marked by symptoms such as high blood pressure, abnormal cardiovascular adaptations in the mother, poor placental vascularization, proteinuria, and restricted fetal growth [1]. PE is a prevalent gestational complication affecting 5%–8% of pregnancies and the leading cause of maternal and fetal morbidity and mortality globally [1–3]. Approximately 76 000 pregnant women and 500 000 fetuses lose their lives each year due to PE and related hypertensive disorders [4, 5]. PE is associated with risk factors such as obesity, preexisting hypertension, advanced maternal age, and gestational diabetes, while oxidative stress, immune system dysfunction, and angiogenic imbalance are considered its primary causes [6]. Due to the limited options for the treatment of PE, the only definitive solution to lower maternal mortality is pregnancy termination. But this approach does not enhance the long-term outcomes [2]. Therefore, it is essential to identify key biomarkers that can aid in understanding the pathogenesis of the disease and serve as potential therapeutic targets.

Recent studies have identified several key genes associated with PE through various integrative bioinformatics and machine learning (ML) approaches. For instance, Gao *et al*. [1] highlighted CADM3 as a potential biomarker through differential expression (DE) and enrichment analyses, while Li *et al*. [2] identified five prognostic genes by combining co-expression networks with multiple ML algorithms. Similarly, Zheng *et al*. [3] proposed F13A1 and SCCPDH as diagnostic candidates after integrating weighted gene co-expression network and ML methods, validated experimentally. Yu *et al*. [7] further expanded the scope by identifying cuproptosis-associated markers using ResNet and Random Forest.

The primary challenge in discovering biomarkers for PE lies in the complexity of the disease and the inability of a single biomarker to provide a reliable diagnosis, particularly in the early stages of pregnancy [3]. Although recent studies have identified numerous genomic biomarkers from gene expression data [2, 3, 7–9], pinpointing crucial biomarkers for PE remains challenging. This difficulty arises due to the high dimensionality of gene expression datasets, leading to the “curse of dimensionality,” which makes it harder to extract significant genes. Additionally, gene expression datasets contain redundant data, as genes with similar expression patterns create redundancy. To tackle these challenges, integrated bioinformatics approaches along with ML and deep learning techniques [10–12] have recently been increasingly applied in biomedical research to efficiently analyze and manage large-scale datasets. ML algorithms such as LASSO, SVM [6], and RF [3], among others, are widely applied to identify and validate key biomarkers from genetic data. Previous studies have demonstrated that Fisher score-based feature selection effectively identifies significant gene subsets from gene expression datasets [13]. Likewise, minimum Redundancy Maximum Relevance (mRMR) has been reported as an effective approach for selecting the most relevant disease-associated genes while eliminating redundancy [14]. Additionally, classifiers play a crucial role in validating the robustness of the discriminatory power of the selected gene subsets [6, 13].

In this study, we proposed a comprehensive approach named AttBiomarker to identify potential biomarkers and therapeutic targets for PE. Initially, we applied the Fisher score [15] to three microarray datasets to create a gene subset based on feature importance. The refined subset underwent further optimization using the mRMR algorithm. These selected features were evaluated using various ML classifiers, including SVM, XGBoost, LightGBM, CNN, and Attention-based CNN, to identify the most effective model for classification. Additionally, we conducted DE analysis to identify significantly expressed genes and performed functional enrichment analyses, including Gene Ontology (GO) and KEGG pathway analyses, to understand the biological processes and pathways associated with PE. We identified hub genes (HGs) from the protein–protein interaction network (PPIN) and carried out gene regulatory network (GRN) analysis. Additionally, molecular docking was performed between the key HGs and two drug compounds to evaluate their binding affinity, aiming to propose potential drug candidates for PE treatment.

**Figure 1:**
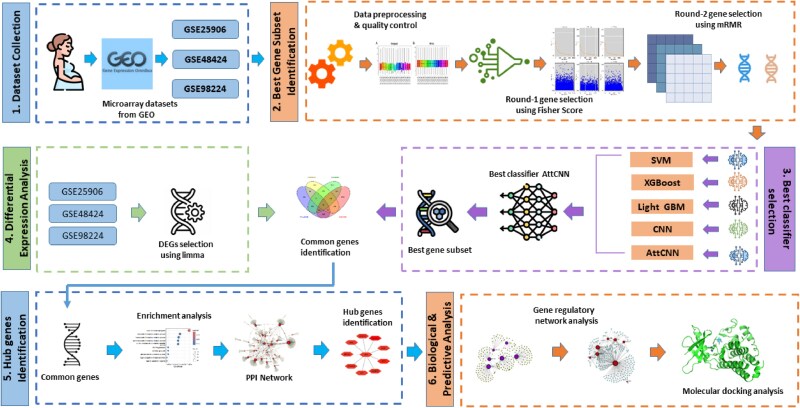
Systematic framework for identifying biomarkers in preeclampsia; (1) Microarray datasets were collected from GEO; (2) best gene subset identification using a two-step feature selection process (Fisher score and mRMR); (3) optimal classifier selection from multiple models, including SVM, XGBoost, LightGBM, CNN, and AttCNN; (4) pinpointing common genes by intersecting DEGs from DE analysis and the selected gene subset; (5) PPIN composition and HGs identification; (6) GRN analysis and molecular docking to assess drug–gene binding affinity.

While most of the previous studies identified promising biomarkers using diverse ML and network-based methods, most rely on traditional feature selection techniques or focus primarily on individual algorithm performance. In contrast, our approach combines the Fisher score and mRMR algorithms to more effectively reduce dimensionality and eliminate redundant features, which addresses a common challenge in gene expression data analysis. Furthermore, we employ an ensemble of classifiers, including both classic ML models and deep learning architectures such as Attention-based Convolutional Neural Network (AttCNN), to capture complex nonlinear relationships in the data. This integrative strategy enables a more robust and comprehensive identification of biomarkers, potentially improving diagnostic accuracy and therapeutic target discovery for PE. The detailed workflow is depicted in Fig. 1 and Algorithm 1.

## Materials and methods

### Data acquisition and quality control

The microarray gene expression data used in this study were obtained from the Gene Expression Omnibus (GEO) database, hosted by the National Center for Biotechnology Information (NCBI) [16]. We retrieved the datasets using the keywords ”Preeclampsia,” ”Preeclamptic pregnancy,” and the organism: *Homo sapiens*. To ensure the biological relevance and consistency of the study, we filtered the datasets to include only those with clearly defined case and control groups and tissue sources directly related to PE, specifically blood and placental samples. We found three microarray datasets for this study, with GEO accession numbers GSE48424 [17], GSE25906 [18], and GSE98224 [19]. The GSE48424 dataset was generated using the GPL6480 platform. This dataset includes gene expression profiles from 38 women, comprising 19 PE patients (13 severe and six non-severe), and 19 gestational age-matched normotensive controls. The background study of the dataset measured circulating gene expression, microparticle release, endothelial responses, and coagulation pathway activity, revealing distinct transcriptional signatures between PE and control groups. The samples were selected based on factors such as gestational age at inclusion, blood pH, race, age, weight, and smoking status [17]. The GSE25906 dataset includes genome-wide gene expression profiles from 60 human placentas to identify gene expression patterns linked to PE [18], while the GSE98224 dataset with the GPL6244 platform integrates gene expression and DNA methylation data from placental samples to investigate regulatory mechanisms underlying PE. Transcriptional analysis identified five placental clusters, with four enriched in PE cases. Epigenetic profiling using Human Methylation 450K arrays provided insights into gene regulation associated with distinct PE subtypes [19]. An overview of these datasets is provided in Table 1. The raw files were preprocessed using background correction with statistical models to eliminate noise and account for nonspecific signals. Robust Multi-Array Average (RMA) normalization [20] was applied to address technical variations across arrays. RMA aggregates probe-level data into gene-level expression values through a median polish algorithm, ensuring consistent and comparable expression values across samples. This method provides more accurate and physiologically relevant results compared with alternatives like MAS5 [13].



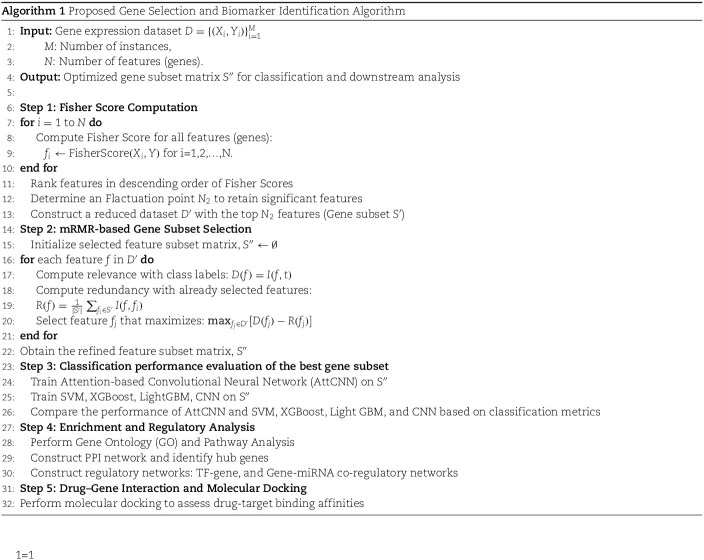



**Table 1:** Datasets description

Dataset	Platform	Preeclampsia	Normal	Total samples
GSE48424	GPL6480	18	18	36
GSE25906	GPL6102	23	37	60
GSE98224	GPL6244	30	18	48

### Gene filtering and matrix transformation

DE analysis was performed using the GEO2R tool from the GEO database [21]. GEO2R utilizes the limma [22] package, which employs empirical Bayes methods to improve the estimation of gene expression variances between groups. This statistical framework produces moderated t-statistics, enabling accurate identification of differentially expressed genes (DEGs). The results from GEO2R were then mapped to the corresponding series matrix, linking gene identifiers in the series matrix to gene symbols and $P$-values obtained from the DEG analysis. The $P$-value reflects the statistical significance of each gene’s DE and serves as a key criterion for selecting significant genes.

A threshold of $ P <.25 $ was applied to retain a substantial number of significantly expressed genes for downstream ML-based feature selection. This threshold was chosen to avoid prematurely excluding potentially informative genes that, while not meeting stricter statistical cutoffs, could still meaningfully contribute to classification performance when considered alongside others or possess underlying biological relevance. Genes with $P$-values exceeding this threshold were excluded, retaining only those with strong evidence for DE. After filtering, the expression values of the selected genes were collected, and the gene expression matrix $S$ was constructed. The matrix $ S $ is structured as follows:


\begin{align*} & S = \begin{pmatrix} d_{11} & d_{12} & \dots & d_{1p} \\ d_{21} & d_{22} & \dots & d_{2p} \\ \vdots & \vdots & \ddots & \vdots \\ d_{q1} & d_{q2} & \dots & d_{qp} \end{pmatrix} \end{align*}

Here, $ S $ represents the gene expression values for $ q $ samples across $ p $ genes, with each row corresponding to the expression profile of an individual sample.

### Two-step feature selection

**Figure 2:**
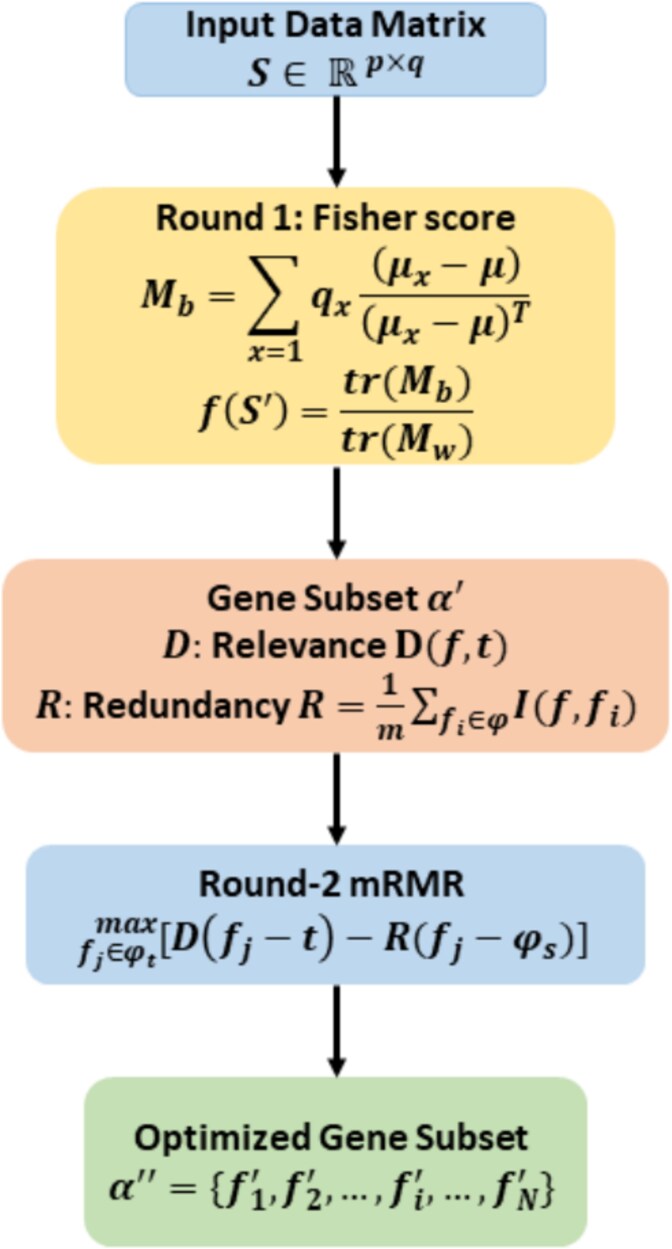
Flowchart of the two-step feature selection approach. The first stage applies Fisher Score to evaluate gene importance and reduce dimensionality, followed by mRMR to refine the subset by maximizing relevance and minimizing redundancy.

Classifying high-dimensional data poses significant challenges due to the curse of dimensionality [23]. Feature selection addresses this issue by eliminating irrelevant or redundant features, and retaining a subset of the most relevant ones to enhance model performance. Noisy and redundant features in high-dimensional datasets can negatively impact the performance of classification models. A two-step feature selection approach helps address this issue by identifying and extracting the most relevant gene subsets, enhancing model accuracy and efficiency [13, 14]. The overall workflow of the two-stage feature selection approach is illustrated in Fig. 2, which summarizes the key steps from initial gene ranking using Fisher Score to subset refinement using the mRMR strategy.

#### First step feature selection using Fisher score

Among feature selection approaches, filter-based methods rank features using statistical metrics independently of the learning algorithm, selecting those with the highest scores [24]. The Fisher score, a well-established filter-based supervised feature selection technique, was employed to assess the importance of each gene.

The principal idea of the Fisher score is to select a subset of features such that, in the data space defined by these features, the separation between data points of different classes is maximized, while the compactness within each class is minimized [25]. This method evaluates each feature independently based on its score calculated under the Fisher criterion. However, this approach may result in a suboptimal feature subset, potentially increasing computational and storage complexity during data processing.

Given the selected $ r $ features, the input data matrix $ S \in \mathbb{R}^{q \times p} $ is transformed into $ S^{\prime} \in \mathbb{R}^{q \times r} $. The Fisher Score (FS) is then calculated as


(1) \begin{align*}& f(S^{\prime}) = \frac{\text{tr}(M_{b})}{\text{tr}(M_{w})},\end{align*}

where $\text{tr}(\cdot )$ denotes the trace of a matrix, $ M_{b} $ is the between-class scatter matrix, and $ M_{w} $ is the within-class scatter matrix, defined as


(2) \begin{align*} & M_{b} = \sum_{x=1}^{l} q_{x} (\bar{\mu}_{x} - \bar{\mu})(\bar{\mu}_{x} - \bar{\mu})^{T} \end{align*}

(3) \begin{align*} & M_{w} = \sum_{j=1}^{q} (z_{j} - \bar{\mu})(z_{j} - \bar{\mu})^{T} \end{align*}

Here, $\bar{\mu }_{x}$ and $q_{x}$ represent the mean vector and the number of samples for the $x$th class in the reduced data space $S^{\prime}$. The overall mean vector $\bar{\mu }$ is given by the weighted sum:


(4) \begin{align*}& \bar{\mu} = \frac{\sum_{x=1}^{l} q_{x} \bar{\mu}_{x}}{\sum_{x=1}^{l} q_{x}}\end{align*}

Let $ \mu _{i}^{x} $ and $ \sigma _{i}^{x} $ represent the mean and standard deviation of samples from the $ x $th class corresponding to the $ i $th feature, respectively. Also, let $ \mu _{i} $ and $ \sigma _{i} $ denote the mean and standard deviation of all samples for the $ i $th feature. The Fisher Score (FS) for the $ i $th feature is calculated as follows [26]:


(5) \begin{align*}& f(i) = \frac{\sum_{x=1}^{L} q_{x} (\mu_{i}^{x} - \mu_{i})^{2}}{\sum_{x=1}^{C} q_{x} (\sigma_{i}^{x})^{2}}\end{align*}

In this study, during the initial gene subset selection phase, we applied the Fisher Score feature selection process to evaluate each feature individually based on its score in the Fisher criterion [13] using the input matrix $ S $ with $ q $ samples and $ p $ genes. Using Equation (5), the Fisher scores $ S_{k} $, where $ k \in \{1, 2, \dots , p\} $, were computed for all genes. As shown in the curve plot of sorted gene scores in Fig. 4, a gene subset $ \alpha ^{\prime} $, consisting of genes selected just after the deflection point, was chosen to construct a reduced gene expression matrix $ S^{\prime} $, ensuring the retention of potentially informative features while significantly reducing the dimensionality of the original matrix.

#### Second step feature selection using mRMR

Usually DEGs are identified using empirical Bayes moderated tests, with adjustments made for the false discovery rate to ensure statistical significance. While this approach effectively identifies a subset of DEG, it does not address potential redundancy among the selected genes. To overcome this limitation, the mRMR method is applied as an optimal feature selection strategy to minimize redundancy while maximizing relevance, thereby improving the quality of the selected gene subset [27]. The mRMR algorithm is a highly reliable feature selection method in ML and has been widely applied in multi-omics medical research in recent years [14].

The mathematical framework for the mRMR algorithm is described as follows [14]. Let $ \Phi $, $ \Phi _{s} $, and $ \Phi _{t} $ denote the set of all features (all genes from $ S^{\prime} $ which were selected applying Fisher score), the set of selected features, and the set of features to be selected, respectively. The relevance ($ D $) of a feature $ f $ from $ \Phi _{t} $ with the target tissue or cell type $ t $ is measured using mutual information ($I $):


(6) \begin{align*}& D = I(f, t)\end{align*}

The redundancy ($ R $) of a feature $ f $ with the features already selected in $ \Phi _{s} $ is defined as


(7) \begin{align*}& R = \frac{1}{m} \sum_{f_{i} \in \Phi_{s}} I(f, f_{i}),\end{align*}

where $ m $ represents the number of features in $ \Phi _{s} $. The objective is to select a feature $ f_{j} $ from $ \Phi _{t} $ that maximizes the relevance $ D $ while minimizing the redundancy $ R $. This optimization problem can be expressed as


(8) \begin{align*}& \max_{f_{j} \in \Phi_{t}} \left[ D(f_{j}, t) - R(f_{j}, \Phi_{s}) \right]\end{align*}

After $ n $ iterations of evaluation, all features ($ \Phi $) are ranked to produce a reordered feature list $ \alpha ^{\prime\prime} $ of the new gene expression matrix of $ S^{\prime\prime} $ as follows:


(9) \begin{align*}& \alpha^{\prime\prime} = \{ f_{1}^{\prime}, f_{2}^{\prime}, \dots, f_{i}^{\prime}, \dots, f_{N}^{\prime} \}\end{align*}

In this list, the index $ i $ indicates the trade-off between the feature’s relevance to the target and its redundancy with the already selected features. A smaller index $ i $ corresponds to a feature with higher discriminative power, resulting in a higher rank for the corresponding feature $ f_{i} $.

### Proposed AttCNN

An AttCNN was proposed for classification using various gene subsets, including the optimal gene subset for PE. AttCNN has demonstrated notable improvements in both performance and interpretability in diverse biomedical studies [28, 29]. The AttCNN model has five key components and was designed specifically to differentiate between normal and PE samples. The mathematical formulations representing the functionality of the different layers within the AttCNN are described as follows [30, 31]:

**(i) Convolution layer:** the convolution operation enables the network to identify localized dependencies between genes. This is particularly important in gene expression data, where certain genes may exhibit co-expression patterns or interact biologically, influencing the overall classification (e.g. normal versus PE samples). The convolution operation is expressed as


(10) \begin{align*}& h_{i} = f(W \cdot X_{i:i+k-1} + b),\end{align*}

where

-
$h_{i}$ is the output of the convolution operation at position $i$.
-
$f$ is the activation function (ReLU in this case, defined as $f(x) = \max (0, x)$).
-
$W$ is the weights of the convolution filter (learned during training).
-
$b$ is the bias term (also learned during training).
-
$X_{i:i+k-1}$ is a sliding window of input features (gene expression values), where $k$ is the kernel size.

Although convolutional layers are traditionally used in image processing, they have also proven beneficial for gene expression data. This is because convolution can effectively capture local dependencies and co-expression patterns among neighboring genes, which may be biologically relevant. Recent studies have demonstrated that CNN-based architectures can outperform classical ML models in biomedical classification tasks by learning hierarchical representations from raw gene data [32, 33]. Thus, the convolutional layer in our AttCNN model is essential for extracting informative and locally structured features from gene expression profiles.

**(ii) Attention mechanism:** the attention mechanism enhances the interpretability of the model by identifying which features (genes highly associated with PE) contribute most significantly to the classification decision. This layer can be described as follows:

**(a) Attention weights** ($\alpha _{i}$): each feature vector $h_{i}$ is assigned an attention weight $\alpha _{i}$, which quantifies its importance. The weights are computed using the following formula:


(11) \begin{align*}& \alpha_{i} = \frac{\exp(u_{i}^\top u_{w})}{\sum_{j=1}^{n} \exp(u_{j}^\top u_{w})},\end{align*}

where

-
$u_{i}$ is a feature vector transformed into an attention space.
-
$u_{w}$ is a learnable attention vector that determines the alignment between the features and the task.
-
$\exp (\cdot )$ is the exponential function that ensures all weights are positive.
- The denominator normalizes the weights to sum to 1, making it a softmax distribution.

**(b) Weighted features** ($c$): the attention weights are used to scale the feature map, creating a context vector $c$, which is a weighted sum of the features:


(12) \begin{align*}& c = \sum_{i=1}^{n} \alpha_{i} h_{i}\end{align*}

Here,

-
$c$ is the final output of the Attention layer, representing the combination of features deemed most important.
-
$\alpha _{i}$ indicates the contribution of $h_{i}$ to $c$.

**(iii) Pooling layer:** pooling layers, such as max-pooling or average-pooling, are used to reduce the dimensions of feature maps produced by convolutional layers. This dimensionality reduction retains the most significant information, simplifying the extracted features and enhancing classification efficiency. Mathematical representation of this layer is


(13) \begin{align*}& g = \frac{1}{n} \sum_{i=1}^{n} h_{i}\end{align*}

-
$g$ is the average of the feature values.
-
$n$ is the total number of elements.
-
$h_{i}$ is the $i$th feature value in the set.

**(iv) Fully connected layer:** it serves as the decision-making layer by mapping the extracted features to the target labels for classification. This layer can be derived by the following equations:


(14) \begin{align*}& z = f(W_{f} \cdot c + b_{f}),\end{align*}

where

-
$z$ is the output of the dense layer (predicted values for classification).
-
$W_{f}$ is the trainable weight matrix.
-
$c$ is the input features (context vector or pooled features).
-
$b_{f}$ is the bias term.

**(v) Output layer:** the last layer of the model computes the weighted total of the CNN outputs generated by the attention mechanism and generates the ultimate classification, either normal or PE sample.


(15) \begin{align*}& \hat{y} = \sigma(W_{o} \cdot z + b_{o}),\end{align*}

where

-
$\hat{y}$ is the predicted probability for the positive class (e.g. PE).
-
$\sigma (x)$ is the sigmoid activation function, defined as $\sigma (x) = \frac{1}{1 + \exp (-x)}$.
-
$W_{o}$ is the weight matrix for the output layer.
-
$z$ is the input feature vector from the previous dense layer.
-
$b_{o}$ is the bias term added to the linear transformation.

Initially, a gene subset was selected through DE analysis based on $P$-values, and the Attention-based CNN (AttCNN) was applied to this feature set as a baseline. Subsequently, the features were incrementally reduced based on their importance, as determined by the Fisher score. Four subsets with varying numbers of genes were generated, and AttCNN was applied to each subset. Performance metrics, including AUC, accuracy (ACC), F1 score, precision (PRE), and recall (REC), were used to evaluate the model’s performance. To ensure robust and generalizable performance evaluation, we employed a five-fold cross-validation strategy during model training and testing. Following the first round of evaluation, the best-performing subset was further refined using the mRMR technique. This process resulted in the creation of four additional subsets, which were then fed into the AttCNN model solely for classification, and their performance was evaluated using the same metrics. After identifying the optimal feature subset, several classifiers—SVM, XGBoost, LightGBM, and CNN—were applied to compare their performance with that of AttCNN, ensuring a comprehensive evaluation of the model’s effectiveness.

### Functional enrichment analysis, PPI network, and GRN analysis

A significant gene subset was identified from three microarray datasets (GSE48424, GSE25906, and GSE98224) using FS-mRMR. To enhance the robustness of the findings, DEGs were independently identified using the limma package in R [22] from the same datasets based on a threshold of $P$-value ¡.05 and $|logFC|> 1$. The common genes between the DEGs from the three datasets and the gene subset obtained through FS-mRMR were then extracted for further analysis.

Gene set enrichment analysis examines gene sets based on their shared biological functions and chromosomal locations. The primary purpose of identifying GO terms is to gain insights into the molecular functions, cellular roles, and subcellular locations where genes carry out their activities. Additionally, the Kyoto Encyclopedia of Genes and Genomes (KEGG) pathway is widely utilized for understanding metabolic pathways and plays a crucial role in gene annotation [34, 35]. Functional enrichment analysis, including GO [36] and KEGG pathway analysis [37], was conducted on the common genes using the clusterProfiler [38] package in R and Enrichr [39]. Statistically significant enrichments were identified based on a threshold of $P <.05$. These analyses revealed the key biological processes, molecular functions, cellular components, and pathways associated with the common genes.

A PPIN network provides a framework for systematically identifying disease-related genes by analyzing the relationships between genes with analogous functionalities [40]. A PPIN for the common genes was constructed using NetworkAnalyst [41] (https://www.networkanalyst.ca/) and subsequently visualized and analyzed in Cytoscape (version 3.10.1). The top 10 HGs were identified using the degree method provided by the cytoHubba [42] plugin.

GRNs establish regulatory relationships between genes, enhancing the understanding of their biological roles, including molecular functions and broader functional activities of individual genes [43]. It is a method used to explore how genes influence and regulate each other’s expression, playing a central role in controlling cellular functions and development. It involves building and examining networks composed of genes, transcription factors (TFs), and other regulatory components to uncover the regulatory mechanisms driving gene expression [44]. We analyzed two types of GRNs: TF–gene (TFG) interactions and gene–microRNA (miRNA) interactions. TFG interactions reveal how genes work together in regulatory networks and pathways [45]. TFG interaction analysis was conducted on the common genes using NetworkAnalyst. The ENCODE database (https://www.encodeproject.org/), integrated within NetworkAnalyst, was utilized to construct the TF–gene network. Gene–miRNA interaction was also performed using the same tool. In addition to using NetworkAnalyst, TF enrichment analysis was conducted using the ChEA3 tool.

### Molecular docking analysis

We performed molecular docking to suggest drug compounds for PE treatment by analyzing the interactions between potential biomarkers and drugs. First, we obtained the 3D structures of target proteins from the Protein Data Bank (PDB) (https://www.rcsb.org/). Using AutoDock Tools (version 1.5.7), we prepared the protein structures by removing water molecules and adding polar hydrogens, then saved them in PDBQT format for docking. Next, we retrieved the molecular structures of methyldopa and labetalol [46], two commonly used drugs for PE, from PubChem in SDF format. These structures were then visualized and converted into the appropriate format using PyMOL (version 3.1.3.1). Finally, molecular docking was performed, and the binding affinities among proteins and ligands were analyzed and visualized in PyMOL, providing insights into potential drug–target interactions.

## Results

### Initial gene filtering

**Figure 3:**
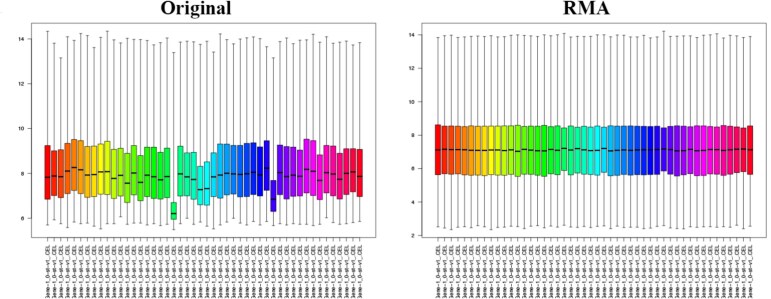
Boxplots representing gene expression distributions before and after RMA normalization. The original gene expression data exhibit varying distributions across samples, indicating potential biases. After RMA normalization, the distributions are more uniform across all samples, ensuring comparability and reducing technical variability in the dataset.

**Figure 4:**
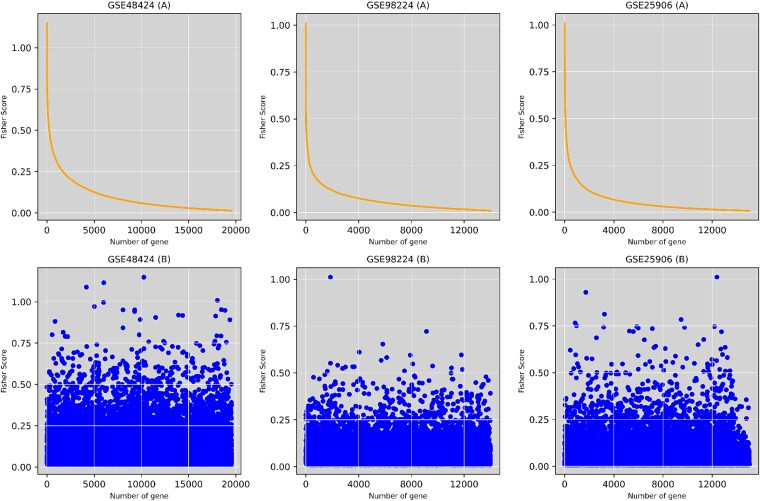
Gene subset selection using Fisher score. The upper row displays the distribution of Fisher scores across genes, where a noticeable deflection point in the curve was visually identified. Genes following this deflection were selected to form an informative subset with reduced dimensionality. The bottom row shows the scatter distribution of Fisher scores for all genes in each dataset.

Three microarray datasets (GSE48424, GSE98224, and GSE25906) were processed for gene filtering, with RMA normalization applied in the initial stage. RMA normalization performed background correction to reduce noise, and quantile normalization to align expression distributions across all samples. As shown in Fig. 3, before normalization, the gene expression data exhibited significant variability in distribution across samples. After applying RMA, the distributions became more uniform, ensuring comparability and reducing technical variability in the dataset. Initial gene filtering was conducted on these microarray datasets to identify significant genes. The filtering was based on a $P$-value threshold of $<0.25$, which was set slightly higher to ensure a sufficient number of genes were retained. This analysis identified 15 021 genes from the GSE25906 dataset, 13 549 from GSE48424, and 13 989 from GSE98224 (Fig. 4). Then, 5334 common genes were identified across the three datasets, and a gene expression matrix $S$ was constructed. This matrix consisted of 145 samples and 5334 genes.

### Effectiveness of dimensionality reduction using Fisher score and mRMR

**Figure 5:**
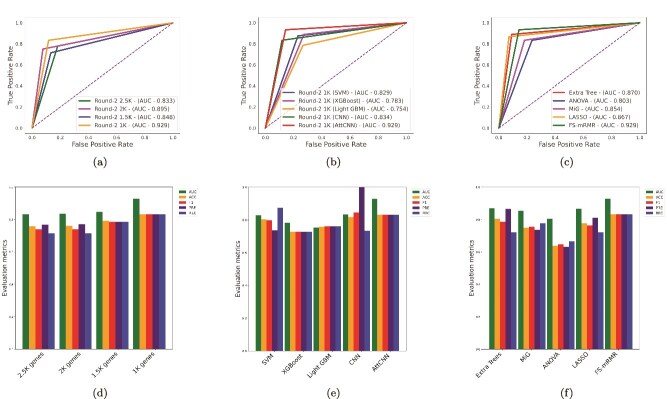
Comparative performance analysis of feature selection and classification methods. (a) ROC curves comparing different gene subsets selected in FS Round 2 using mRMR. (b) ROC curves illustrating the classification performance of different ML models on the final subset of 1000 genes. (c) ROC curves demonstrating the impact of various feature selection methods. (d) Bar chart showing evaluation metrics (AUC, ACC, F1, Precision, Recall) for different gene subsets from FS Round 2. (e) Performance comparison of different classifiers on the final gene subset. (f) Evaluation metrics for various feature selection techniques, highlighting FS-mRMR as the most effective approach.

Feature selection techniques were employed to identify the optimal gene subset from our three high-dimensional microarray datasets. In the first step, an initial gene set was selected without feature selection. Subsequently, multiple refined gene subsets were generated through systematic two-step feature selection. Each subset was then fed into our proposed AttCNN model, assessing its classification performance based on accuracy, AUC, F1-score, precision, and recall. The identification of various gene subsets and their corresponding classification efficiency using AttCNN are summarized in Table 2, Fig. 5(a),(d), and Supplementary Fig. S1.

**Table 2:** Performance evaluation of the gene subsets using AttCNN

	Feature	AUC	ACC	F1 score	PRE	REC
**Without FS**	5334	0.7095	62.06%	0.5611	0.6362	0.5232
**FS Round 1**	5000 Sub 1	0.7121	65.51%	0.6667	0.6251	0.7142
	4500 Sub 2	0.7714	66.01%	0.6875	0.6111	0.7857
**Fisher Score**	4000 Sub 3	0.7963	68.96%	0.6667	0.6922	0.6428
	**3500 Sub 4**	0.7571	69.23%	0.6421	0.7272	0.6428
**FS Round 2**	2500 Sub 1	0.8333	75.86%	0.7407	0.7692	0.7142
	2000 Sub 2	0.8951	76.12%	0.7415	0.7721	0.7142
**mRMR**	1500 Sub 3	0.8476	79.31%	0.7857	0.7857	0.7857
	**1000 Sub 4**	0.9290	83.33%	0.8333	0.8333	0.8333

**FS**, feature selection.

Initially, we used the gene expression matrix containing 5334 genes without applying feature selection. As shown in Table 2, this subset achieved an accuracy of 62.06% and an AUC of 0.7095. Then, in our round 1 of feature selection, we applied the Fisher score method across three datasets to rank genes based on their discriminative power. The Fisher score measures the ratio of between-class variance to within-class variance, allowing us to prioritize genes that best distinguish between conditions. We visualized the Fisher scores as a function of the number of genes (Fig. 4) and determined the subset size by identifying the deflection point where the curve begins to plateau. Using visual inspection, we selected four gene subsets comprising 5000, 4500, 4000, and 3500 genes. In this round, the 3500-gene subset demonstrated the highest accuracy of 69.23%, whereas the 5000-gene subset achieved the lowest accuracy of 65.51%. This marks an improvement of 7.17% and 3.45%, respectively, compared with the initial 5334-gene subset without feature selection. The subsets with 4500 and 4000 genes obtained moderate accuracy, falling between the performance of the 5000-gene and 3500-gene subsets. The final subset of this round was then carried forward for further analysis to ensure the inclusion of the most informative genes.

To enhance the robustness of identifying the most significant gene subset, a secondary feature selection algorithm, mRMR, was applied to the 3500 genes obtained from round 1, and four subsets consisting of 2500, 2000, 1500, and 1000 genes were subsequently identified. As shown in Table 2 and Fig. 5(a),(d), in round 2, the final gene subset with 1000 genes achieved the highest accuracy of 83.33%, while the subset with 2500 genes attained 75.86%, showing a 7.47% increase (7.21% and 4.02% higher than the other two subsets, respectively). Overall, the top-performing subsets from each feature selection round demonstrated accuracy improvements of 7.17% and 21.27%, respectively, compared with the initial 5334-gene subset without feature selection.

To evaluate the effectiveness of the FS-mRMR method, we compared its performance with other feature selection techniques, including Extra Trees [47], MiG [48], ANOVA [49], and LASSO [50]. For each method, distinct gene subsets were identified and evaluated using proposed AttCNN. As shown in Table 3, Supplementary Table S1, and Fig. 5(c),(f), the FS-mRMR method, which selected a subset of 1000 genes, achieved superior performance with an AUC of 0.9290, accuracy of 83.33%, and an F1-score, precision, and recall of 0.8333 each. The Extra Trees method ranked second, achieving an accuracy of 80.55% and an AUC of 0.8703, followed by LASSO, which secured third place with an accuracy of 77.78% and an AUC of 0.867. Other methods, including MiG and ANOVA, showed relatively lower performance, with ANOVA having the lowest accuracy of 63.88%. In addition, we conducted a statistical comparison using the DeLong test to assess the differences in AUC between FS-mRMR and other feature selection methods. As depicted in Table 4, FS-mRMR significantly outperformed ANOVA ($P$ = 0.003); however, its performance differences with Extra Trees, MiG, and LASSO were not statistically significant. Despite this, FS-mRMR uniquely integrates both feature relevance and redundancy minimization, distinguishing it from conventional univariate methods. This dual consideration can lead to more compact and biologically meaningful gene subsets, thereby enhancing downstream interpretability and biological validation. Therefore, even with marginal statistical differences, FS-mRMR remains advantageous for feature selection in high-dimensional gene expression data.

**Table 3:** Performance comparison of FS-mRMR with other feature selection methods

Feature selection	AUC	ACC	F1 score	PRE	REC
Extra Trees	0.8703	80.55%	0.7878	0.8666	0.7222
MiG	0.8546	75.11%	0.7564	0.7368	0.7781
ANOVA	0.8051	63.88%	0.6486	0.6315	0.667
LASSO	0.867	77.78%	0.764	0.812	0.7223
**FS-mRMR**	**0.9290**	**83.33%**	**0.8333**	**0.8333**	**0.8333**

**FS**, Fisher score.

**Table 4:** Statistical comparison of AUC scores between FS-mRMR and other feature selection methods using the DeLong test

Comparison	$\Delta $ AUC	Z-statistic	$P$ -value
FS-mRMR versus Extra Trees	0.0587	1.442	.149
FS-mRMR versus MiG	0.075	1.829	.067
FS-mRMR versus ANOVA	0.124	2.967	.003
FS-mRMR versus LASSO	0.062	1.520	.128

### Classification efficiency evaluation of gene subsets using AttCNN

The classification performance of gene subsets was evaluated using an AttCNN. The AttCNN architecture comprised two convolutional layers with 32 and 64 filters (kernel size = 3, ReLU activation), followed by global average pooling and an attention mechanism. The resulting features were passed through a fully connected layer with 128 neurons (ReLU activation) and a dropout rate of 0.5, ending with a sigmoid-activated output layer for binary classification. The model was trained using the Adam optimizer with binary cross-entropy loss and contained 8263 593 trainable parameters. The dataset was initially split into 80% training and 20% testing using stratified sampling to preserve class distribution. To ensure robustness and generalizability, five-fold cross-validation was applied to the training set. The model was trained for up to 50 epochs with a batch size of 16. Early stopping was implemented based on validation loss with a patience of five epochs, and the best-performing weights were restored. Hyperparameters, including learning rate, dropout rate, and number of filters, were optimized using grid search across the cross-validation folds. Final model performance was evaluated on the test set using accuracy, precision, recall, F1-score, and AUC-ROC metrics.

In this section, we also evaluated the performance of our proposed AttCNN model in comparison with other classifiers, including SVM, XGBoost, LightGBM, and CNN. All of these models were trained on different gene subsets identified through various feature selection techniques, including our FS-mRMR approach. The classification efficacy for the selected subsets are detailed in Supplementary Tables S1–S5 and Supplementary Fig. S3. Table 5 and Fig. 5 (along with Supplementary Fig. S2) illustrate the classification performance of AttCNN and the other classifiers across three scenarios: gene subset without feature selection, the best gene subset from round 1, and the best gene subset from round 2.

**Table 5:** Performance comparison of AttCNN with other classifiers

	Method	AUC	ACC	F1 score	PRE	REC
Without FS	SVM	0.7582	59.09%	0.6244	0.6818	0.5769
	XGBoost	0.7293	61.90%	0.6667	0.6810	0.6521
	LightGBM	0.7582	59.09%	0.5912	0.6111	0.6103
	CNN	0.7115	58.62%	0.5384	0.5384	0.5384
	**AttCNN**	**0.7095**	**62.06%**	**0.5612**	**0.6363**	**0.5232**
FS Round 1	SVM	0.7952	63.88%	0.6829	0.7368	0.6363
	XGBoost	0.7193	65.91%	0.6341	0.5912	0.6843
Fisher Score	LightGBM	0.7647	63.64%	0.6435	0.6515	0.6624
	CNN	0.7252	68.96%	0.5715	0.4613	0.7514
	**AttCNN**	**0.7571**	**69.23%**	**0.6426**	**0.7272**	**0.6428**
FS Round 2	SVM	0.8297	80.55%	0.7991	0.7368	0.8752
	XGBoost	0.7835	72.73%	0.7272	0.7272	0.7272
mRMR	LightGBM	0.7548	75.86%	0.7627	0.7617	0.7612
	CNN	0.8343	81.81%	0.8462	1.000	0.7333
	**AttCNN**	**0.9290**	**83.33%**	**0.8333**	**0.8333**	**0.8333**

**FS**, feature selection.

As presented in Table 5, for the gene subset without feature selection, CNN exhibited the lowest accuracy at 58.62%, whereas AttCNN achieved the highest accuracy of 62.06%. This corresponds to an improvement of 3.44% over CNN, 2.97% over both SVM and LightGBM, and 0.16% over XGBoost. When evaluating the best gene subset from round 1, AttCNN again demonstrated superior performance with the highest accuracy of 69.23%. CNN secured the second position, followed by XGBoost in third, while LightGBM recorded the lowest accuracy, trailing AttCNN by 0.27%, 3.32%, and 5.59%, respectively. For the final and optimal gene subset from round 2, as presented in Table 5 and Fig. 5(b), (e), AttCNN achieved the highest accuracy of 83.33% and an AUC of 0.9290. At this stage, AttCNN again outperformed the other classifiers, surpassing SVM by 2.78%, XGBoost by 10.6%, LightGBM by 7.47%, and CNN by 1.52%.

To further evaluate the contribution of different components within the AttCNN model, an ablation study was conducted, where key elements such as attention layers and fully connected layers were systematically removed. The detailed results of the ablation study are summarized in Table 6. The baseline model, which included all layers, achieved an accuracy of 83.33% and an AUC score of 0.9290. Removing either the first (WFAL) or second attention layer (WSAL) resulted in a slight decrease (8.33% and 5.55%, respectively) in performance, both achieving accuracy of 75.00% and 77.78%, correspondingly. However, when both attention layers were removed (RBAL), a more significant drop in accuracy of 11.11% was observed, reducing the accuracy to 72.22%. Interestingly, removing the fully connected layer (RFCL) did not significantly degrade performance, maintaining an accuracy of 77.78%. These findings reinforce the importance of the attention mechanism in improving the model’s classification performance. Thus, AttCNN emerged as the best classifier in our study.

**Table 6:** Ablation study results of AttCNN

Model	Accuracy	Precision	Recall	F1 Score	AUC Score
Without First Attention (WFAL)	75.00%	1.0000	0.5000	0.6667	0.9167
Without Second Attention (WSAL)	77.78%	0.7778	0.7778	0.7778	0.8704
Remove Both Attention (RBAL)	72.22%	0.7500	0.6667	0.7059	0.8673
Remove Fully Connected Layer (RFCL)	77.78%	0.7778	0.7778	0.7778	0.9012
**Baseline**	**83.33%**	**0.8333**	**0.8333**	**0.8333**	**0.9290**

### DEGs identification and enrichment analysis

**Figure 6:**
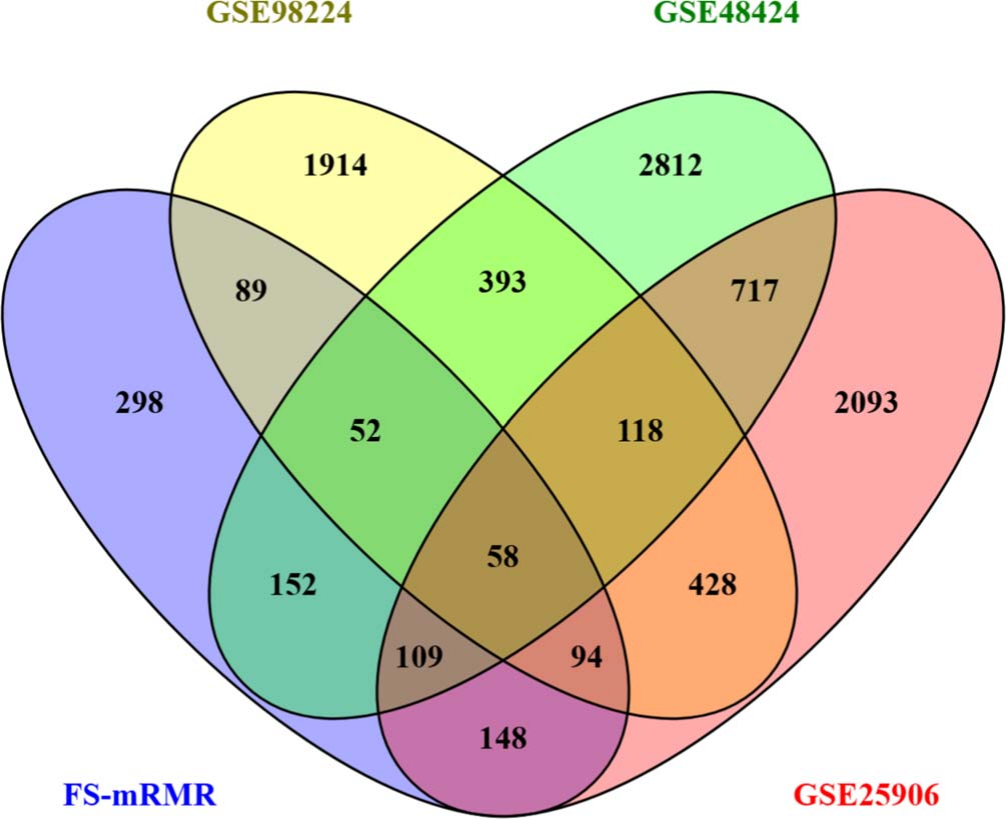
Venn diagram illustrating the overlap between DEGs identified through a systems biology-based DE analysis and the gene subset selected using the FS-mRMR feature selection approach.

DEGs were identified from our three microarray datasets for PE using the limma package in R. The thresholds were set at adjusted $P$-value ¡.05 and $|logFC|> 1$. From the GSE25906 dataset, 3765 DEGs were identified. Among them, 2067 were upregulated, and 1698 were downregulated. The GSE48424 dataset had 4412 DEGs, with 2672 upregulated and 1740 downregulated. The GSE98224 dataset identified 3147 DEGs, including 1912 upregulated, and 1235 downregulated. Common genes were found between these DEGs and the 1000 genes selected through FS-mRMR. A total of 58 common genes were identified, as shown in Fig. 6.

**Figure 7:**
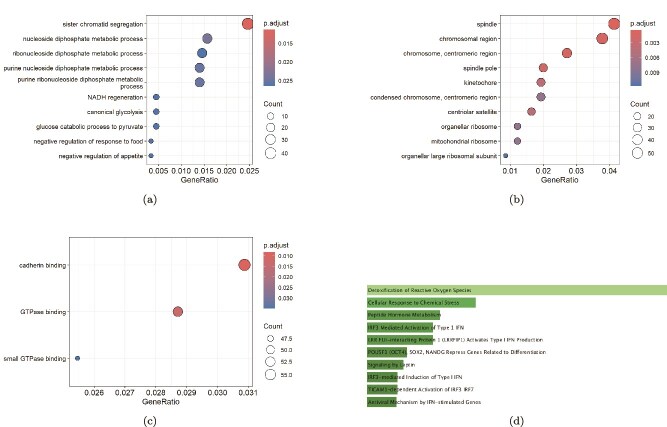
GO and KEGG pathway analysis of the common genes: (a) BP category; (b) CC category; (c) MF category—which provides insights into the biological roles, subcellular localization, and molecular activities of the common genes; (d) KEGG pathway analysis reveals the significant involvement of these genes in various biological pathways.

The 58 common genes were subjected to enrichment analysis using the clusterProfiler package in R. GO analysis revealed significant enrichment across various biological processes (BP), molecular functions (MF), and cellular components (CC). In the BP category, as shown in Fig. 7(a), the genes were significantly enriched in processes such as purine nucleoside diphosphate metabolic process, ribonucleotide diphosphate metabolic process, sister chromatid segregation, and nucleoside diphosphate metabolic process. In the CC category, illustrated in Fig. 7(b), the genes were enriched in terms like spindle, spindle pole, chromosome centromeric region, chromosomal region, and kinetochore. In the MF category (Fig. 7(c)), the genes demonstrated enrichment in cadherin binding, GTPase binding, and small GTPase binding.

KEGG pathway analysis was conducted using Enrichr, revealing that the common genes were significantly enriched in pathways (Fig. 7(d)) such as cellular response to chemical stress, detoxification of reactive oxygen species, peptide hormone metabolism, and IRF3-mediated activation of type 1 IFN, among others.

### P‌PI and GRNs analysis

**Figure 8:**
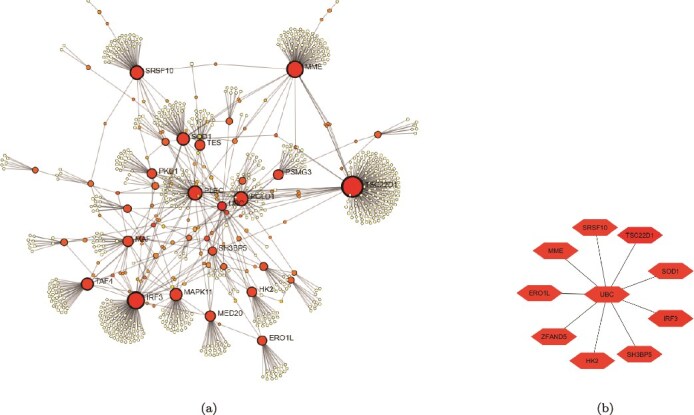
PPI network construction and HG identification. (a) The PPI network consists of 502 nodes and 530 edges. The size and color intensity of the nodes in the network reflect their degree of connectivity (number of interactions), highlighting the central genes in the network. (b) Top 10 HGs identified using degree centrality.

**Figure 9:**
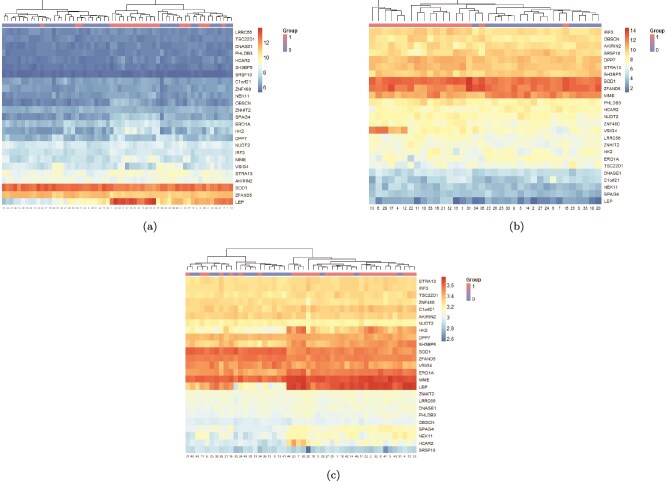
Heatmaps demonstrating the expression profiles of 25 selected genes across three microarray datasets: (a) GSE25906, (b) GSE48424, and (c) GSE98224. The rows represent genes, and the columns represent samples. Expression intensity is shown on a continuous scale from low to high values. Group 1 denotes PE samples; Group 0 represents healthy controls.

A PPIN was constructed with the common genes using NetworkAnalyst. The network, shown in Fig. 8(a), consisted of 502 nodes and 530 edges. It was further visualized and analyzed in Cytoscape. Using the degree method from the cytoHubba plugin, the top 10 HGs were identified. These genes, presented in Fig. 8(b), are TSC22D1, IRF3, MME, SRSF10, SOD1, HK2, ERO1L, SH3BP5, UBC, and ZFAND5. To visualize the expression patterns of the most informative genes, we constructed heatmaps for 25 selected genes, chosen from the intersection of DEGs and FS-mRMR selected genes. The heatmaps in Fig. 9 revealed distinct expression profiles between PE (Group 1) and control (Group 0) samples. Across all three datasets, most genes exhibit consistent DE trends. For instance, IRF3, MME, HK2, and SOD1 are consistently upregulated in PE samples, whereas SRSF10 shows a downregulated pattern. Additionally, the hierarchical clustering illustrates a clear separation between PE and control groups, underscoring the discriminative power of the selected genes.

**Figure 10:**
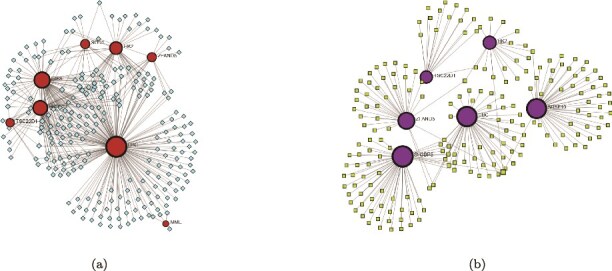
GRN analysis. (a) The TFG interaction network consists of 246 nodes and 412 edges. The smaller nodes represent TF genes, while the bigger nodes indicate HGs. (b) The gene–miRNA interaction network contains 204 nodes and 213 edges, with the smaller nodes representing miRNAs and larger nodes denoting HGs.

**Figure 11:**
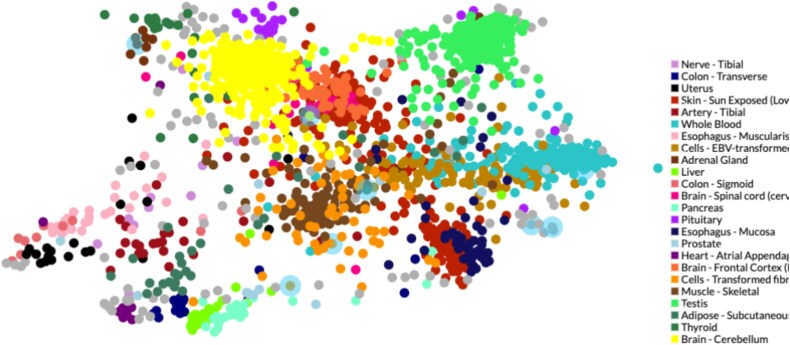
Tissue distribution of TF targets associated with the identified 10 HGs. Each dot represents a TF, and the clustering is based on the similarity of TFs and their associated gene regulatory signatures.

We used the NetworkAnalyst tool to analyze GRNs for the 10 HGs. Two types of networks were studied: the TFG interaction network and the gene–miRNA interaction network. The TFG interaction network, shown in Fig. 10(a), comprised 246 nodes and 412 edges. The top five HGs that interacted were UBC, IRF3, SRSF10, HK2, and SOD1. The distribution of dots in Fig. 11 suggests tissue-specific TF activity. TFs enriched in Nerve—Tibial, Liver, and Esophagus—Mucosa appear clustered together. The gene–miRNA interaction network, shown in Fig. 10(b), consisted of 204 nodes and 213 edges. Six HGs: UBC, SH3BP5, ZFAND5, HK2, TSC22D1, and SRSF10 interacted with various miRNA.

### Molecular docking analysis

**Figure 12:**
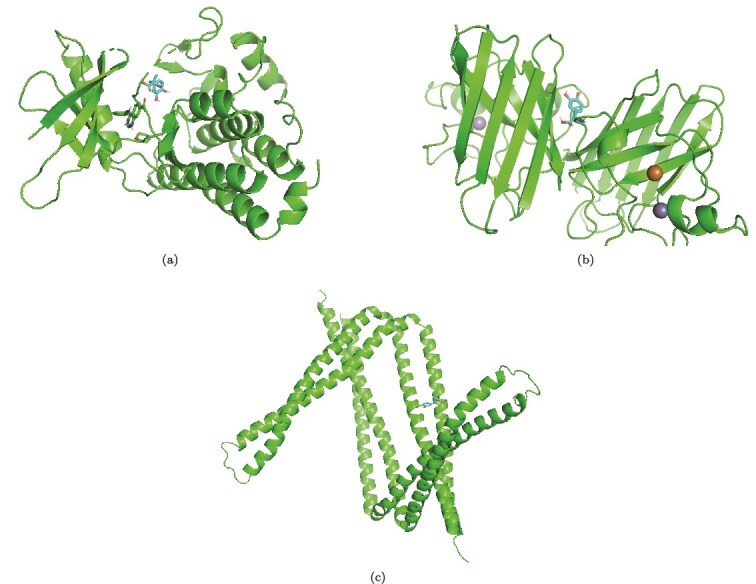
Molecular docking configurations of the three prominent HGs with the Methyldopa drug compound. (a) HK2, (b) SOD1, (c) SH3BP5. Protein structures and ligands are displayed to illustrate the binding regions and conformational fit of Methyldopa within each protein’s active site.

Molecular docking was conducted on the identified HGs and two FDA-approved drugs for PE, methyldopa, and labetalol. The docking analysis assessed the binding affinities of these drugs with the selected target proteins. Binding energy thresholds were considered to evaluate interaction strength: values below −4.25 kcal/mol indicate potential binding activity, values below −5.0 kcal/mol suggest good binding affinity, and values below −7.0 kcal/mol represent strong binding interactions [51]. Among the interactions, SOD1 exhibited the strongest binding affinity with both methyldopa (−6.2 kcal/mol) and labetalol (−7.0 kcal/mol), indicating a stable interaction. HK2 showed binding energies of −5.2 kcal/mol with methyldopa and −6.1 kcal/mol with labetalol. Similarly, SH3BP5 demonstrated binding affinities of −4.9 and −5.5 kcal/mol with methyldopa and labetalol, respectively. These findings highlight potential drug–target interactions that may be relevant for PE treatment. The binding energy values are summarized in Table 7, and the binding configurations are illustrated in Fig. 12 and Supplementary Fig. S4.

**Table 7:** Molecular docking analysis

Gene	Drug compound	Binding energy (kcal/mol)
HK2	Methyldopa	−5.2
HK2	Labetalol	−6.1
SH3BP5	Methyldopa	−4.9
SH3BP5	Labetalol	−5.5
SOD1	Methyldopa	−6.2
SOD1	Labetalol	−7.0

## Discussion

PE is a prevalent hypertensive disorder during pregnancy and a leading cause of complications and mortality during the gestational period [8]. Approximately 76 000 women lose their lives to PE each year, accounting for 16% of maternal deaths worldwide, with the majority occurring in developing countries [8]. Yet, the molecular mechanisms underlying PE remain largely unclear, limiting the opportunities for early prognosis, diagnosis, and treatment options. Significant biomarkers can play a crucial role in the early detection, diagnosis, and treatment of PE. This study focuses on identifying significant biomarkers associated with PE using integrated ML and bioinformatics approaches. Initially, genes were filtered from three microarray datasets based on $P$-values. In the first round of feature selection, the Fisher score was applied to generate four gene subsets. In the second round, the mRMR method was used to create another four gene subsets. Each subset from every round was analyzed using AttCNN to evaluate their discriminative accuracy. The subset containing 1000 genes from Round 2 (FS-mRMR) achieved the highest accuracy. The performance of FS-mRMR in AttCNN was compared with other classifiers, including SVM, LightGBM, XGBoost, and CNN. Additionally, AttCNN was applied to gene subsets obtained from other feature selection methods, such as Extra Tree, MiG, LASSO, and ANOVA. The FS-mRMR-selected genes consistently outperformed all other subsets. In parallel, DEGs were identified from the three microarray datasets. Among these, 58 common genes were identified by intersecting the DEGs with the gene subset selected through FS-mRMR.

Enrichment analysis offers crucial mechanistic insights into gene sets obtained from genetic data analysis, identifying biological pathways that are significantly linked to the given gene set [52]. Functional enrichment analysis, including GO and KEGG pathway analysis, was performed on these common genes. In the BP category of GO, the shared genes were enriched in processes such as sister chromatid segregation, nucleoside diphosphate metabolic process, and ribonucleoside diphosphate metabolic process. In the CC category, the 58 common genes showed significant enrichment in the spindle, chromosomal region, chromosome, and centromeric region. Within the MF category, notable enrichment was observed in cadherin binding and GTPase binding. Additionally, pathway analysis demonstrated that the shared genes were enriched in the detoxification of reactive oxygen species, cellular response to chemical stress, and peptide hormone metabolism.

A PPIN provides deeper insights into the pathogenic mechanisms driving disease onset and progression, enabling the development of effective diagnostic and therapeutic strategies [40]. A PPIN was constructed with the shared genes, and 10 HGs (TSC22D1, IRF3, MME, SRSF10, SOD1, HK2, ERO1L, SH3BP5, UBC, and ZFAND5) were identified.

Hexokinase 2 (HK2) is a key enzyme in glycolysis [53]. It catalyzes a rate-limiting step in aerobic glycolysis by phosphorylating glucose to produce glucose-6-phosphate [54]. A study revealed that HK2P1 and HK2 enhance glucose uptake and lactate production in human endometrial stromal cells. Moreover, both HK2P1 and HK2 are critical for endometrial decidualization and may contribute to the development and progression of PE [53]. HK2 has been linked to several carcinomas, including pancreatic cancer, cervical cancer, glioblastoma, and PE [53–55].

Serine/arginine splicing factor 10 (SRSF10) belongs to the family of mammalian splicing regulators known as SR proteins. Similar to other members of this family, SRSF10 consists of an RNA-binding domain and arginine- and serine-rich auxiliary domains (RS), which facilitate interactions with other proteins. SRSF10 has also been linked to various carcinomas [56]. Superoxide dismutase 1 (SOD1) has been associated with PE [57].

Endoplasmic reticulum oxidoreductase 1 alpha (ERO1L) is an ER luminal glycoprotein involved in forming disulfide bonds in secreted and membrane proteins [58]. It has been linked to PE [59] and various carcinomas, including pancreatic ductal adenocarcinoma (PDAC) [58] and lung adenocarcinoma (LUAD) [60].

SH3BP5 is a key protein involved in immune system regulation and cell signaling. It contains SH3 domains that facilitate PPIs, aiding in signal transduction. SH3BP5 plays a critical role in activating T and B cells, influencing T cell receptor signaling, and regulating immune responses [61]. Its overexpression has been linked to PE and various carcinomas, including lung cancer [61–63].

Among the identified HGs, Interferon Regulatory Factor 3 (IRF3) has also shown a strong association with PE. Previous studies have demonstrated that IRF3 is negatively regulated by miR-92a, which is significantly downregulated in decidual stromal cells of PE patients. The loss of miR-92a leads to overexpression of IRF3, resulting in increased secretion of pro-inflammatory cytokines such as CXL8, CCL5, CXCL3, CXCL2, and IL-6. This cytokine upregulation promotes M1 macrophage polarization, an inflammatory phenotype that has been observed at higher levels in the placental environment of PE cases [64]. IRF3, activated downstream of TLR3, plays a key role in PE by promoting excessive inflammation through interferon-$\beta $ production and contributing to endothelial dysfunction [65]. Its involvement in innate immune activation supports its identification as a HG in our study.

The Membrane Metalloendopeptidase (MME) gene, situated on chromosome 3q21–27 in humans, encodes a 100-kDa transmembrane glycoprotein with its catalytic site positioned on the extracellular side [66]. It was identified as both a differentially methylated and DEG, with its expression upregulated in PE tissues [67]. Additionally, MME has been reported to be associated with other carcinomas, such as esophageal squamous cell carcinoma (ESCC) [68] and breast cancer (BRCA) [66].

GRNs play a crucial role of understaning in defining, sustaining, and dysregulating cellular identity in disease [69]. Our identified HGs demonstrated significant interactions in the TFG interaction network and the gene–miRNA network. Additionally, molecular docking demonstrated the potential binding affinity of HK2, SH3BP5, and SOD1 with two FDA-approved drugs for PE, indicating their possible therapeutic relevance. Notably, previous studies have shown that HK2 exhibits prominent binding affinities (ranging from –5.3 to –8.9 kcal/mol) with natural compounds such as Berberine, Baicalein, and Luteolin, further highlighting its potential as a druggable target in PE. [55].

Seven of the identified HGs (HK2, SRSF10, SOD1, ERO1L, IRF3, MME, and SH3BP5) were associated with PE and various carcinomas. They demonstrated significant interactions with TFs, and miRNAs. Three of them showed significant binding affinity with two drug compounds. These findings highlight that these genes may serve as potential biomarkers for early prognosis, diagnosis, and therapeutic targets in PE.

## Conclusion

Our study systematically investigated the molecular landscape of PE through an integrated bioinformatics and deep learning approach. Gene subset selection was performed using the Fisher score and mRMR, while attention-based CNN was utilized to evaluate the robustness of the selected genes. We identified 58 common genes between ML-based gene selection and DE analysis, which were subjected to enrichment analysis and PPI network construction to determine key molecular interactions. Further analysis led to the identification of HGs, which play critical roles in PE-related biological pathways and disease mechanisms. Besides, functional enrichment analysis revealed significant associations with various biological mechanisms. Additionally, TFG and gene–miRNA regulatory networks were analyzed, providing deeper insights into gene regulation and disease progression. Here, molecular docking analysis provided prominent binding affinity among HGs and drug compounds for PE. The findings of this study may contribute to the growing understanding of PE, offering new perspectives on biomarker identification and disease mechanisms. As PE remains a major cause of maternal and fetal complications, the identified genes and drug compounds from our study could serve as potential diagnostic markers and therapeutic targets, paving the way for more precise and effective treatment strategies for PE. In the future, we aim to extend our analysis to larger and more diverse datasets, including RNA-seq data, and to apply more advanced ML and deep learning methodologies. Additionally, we plan to incorporate bioinformatics tools such as Weighted Gene Co-expression Network Analysis (WGCNA) to further enhance gene network analysis. Experimental validation in the wet lab would be essential to confirm the clinical relevance of these genes and support their potential as diagnostic or therapeutic biomarkers.

**Key Points**

- Developed an integrative deep learning and bioinformatics approach to identify biomarkers for preeclampsia, using microarray datasets and statistical feature selection methods (Fisher Score, mRMR).
- Proposed an Attention-based Convolutional Neural Network (AttCNN), achieving the highest classification accuracy among tested models.
- Identified 58 common genes through differential expression and feature optimization, with enrichment analyses highlighting biological processes and pathways related to preeclampsia.
- Constructed a protein–protein interaction (PPI) network, identifying 10 hub genes, seven of which (HK2, SRSF10, SOD1, ERO1L, IRF3, MME, and SH3BP5) were strongly linked to preeclampsia.
- Molecular docking analysis showed significant drug-binding affinities for HK2, SH3BP5, and SOD1, indicating their potential as therapeutic targets.

Conflict of interest: The authors declare no conflicts of interest.

## Supplementary Material

Supplementary_Files_bbaf473

## Data Availability

The datasets used in this study are publicly available in the Gene Expression Omnibus (https://www.ncbi.nlm.nih.gov/geo/).
