# Supplementary material for: AttBiomarker: unveiling preeclampsia biomarkers and molecular pathways through two-stage gene selection techniques and attention-based CNN with gene regulatory network analysis
Source: Brief Bioinform. 2025 Sep 16;26(5):bbaf473. doi: 10.1093/bib/bbaf473 (PMC12448737; doi:10.1093/bib/bbaf473)
Supplement: Supplementary_Files_bbaf473 [file supplementary_files_bbaf473.docx]

1. **Supplementary Tables**

**Table S1:** Performance evaluation of various feature selections in various classifiers

|  | **Method** | **AUC** | **ACC** | **F1 score** | **PRE** | **REC** |
| --- | --- | --- | --- | --- | --- | --- |
| Extra Trees | SVM | 0.8030 | 72.41% | 0.6363 | 0.6363 | 0.6363 |
|  | XGBoost | 0.7809 | 70.45% | 0.6829 | 0.6363 | 0.7368 |
|  | Light GBM | 0.8654 | 75.86% | 0.7601 | 0.8510 | 0.6905 |
|  | CNN | 0.7212 | 68.96% | 0.6667 | 0.6923 | 0.6428 |
|  | **AttCNN** | **0.8703** | **80.55%** | **0.7871** | **0.8662** | **0.7222** |
| MiG | SVM | 0.7323 | 68.96% | 0.6986 | 0.6363 | 0.5833 |
|  | XGBoost | 0.7809 | 72.73% | 0.7273 | 0.7273 | 0.7273 |
|  | Light GBM | 0.8462 | 75.86% | 0.7586 | 0.7704 | 0.7704 |
|  | CNN | 0.7884 | 74.86% | 0.7407 | 0.7692 | 0.7142 |
|  | **AttCNN** | **0.8543** | **75.11%** | **0.7561** | **0.7368** | **0.7784** |
| ANOVA | SVM | 0.7026 | 55.55% | 0.5294 | 0.4857 | 0.6923 |
|  | XGBoost | 0.7066 | 63.63% | 0.6363 | 0.6363 | 0.6363 |
|  | Light GBM | 0.8061 | 63.52% | 0.6414 | 0.6712 | 0.6712 |
|  | CNN | 0.7256 | 62.06% | 0.6857 | 0.7571 | 0.5714 |
|  | **AttCNN** | **0.8050** | **63.88%** | **0.6486** | **0.6315** | **0.6673** |
| LASSO | SVM | 0.7926 | 58.33% | 0.5454 | 0.6923 | 0.4500 |
|  | XGBoost | 0.8202 | 70.45% | 0.6976 | 0.6818 | 0.7142 |
|  | Light GBM | 0.8317 | 73.65% | 0.7423 | 0.7627 | 0.7314 |
|  | CNN | 0.7523 | 72.41% | 0.6923 | 0.6428 | 0.7510 |
|  | **AttCNN** | **0.8672** | **77.78%** | **0.7644** | **0.8121** | **0.7222** |
| FS-mRMR | SVM | 0.8297 | 80.55% | 0.799 | 0.7368 | 0.875 |
|  | XGBoost | 0.783 | 72.73% | 0.727 | 0.727 | 0.727 |
|  | Light GBM | 0.7548 | 75.86% | 0.762 | 0.761 | 0.761 |
|  | CNN | 0.834 | 81.81% | 0.846 | 1.00 | 0.733 |
|  | **AttCNN** | **0.9290** | **83.33%** | **0.8333** | **0.8333** | **0.8333** |

**Table S2:** Performance evaluation of various subsets using SVM

|  | Feature | AUC | ACC | F1 score | PRE | REC |
| --- | --- | --- | --- | --- | --- | --- |
| Without FS | 5334 | 0.7582 | 59.09% | 0.6244 | 0.6818 | 0.5769 |
| FS Round 1  FS | 5000 Sub 1 | 0.7174 | 52.77% | 0.5142 | 0.6101 | 0.4513 |
|  | 4500 Sub 2 | 0.7826 | 55.55% | 0.5294 | 0.6923 | 0.4285 |
|  | 4000 Sub 3 | 0.7650 | 61.11% | 0.6500 | 0.6190 | 0.6842 |
|  | 3500 Sub 4 | 0.7952 | 63.88% | 0.6829 | 0.7369 | 0.6363 |
| FS Round 2  mRMR | 2500 Sub 1 | 0.7125 | 61.11% | 0.5882 | 0.6250 | 0.5556 |
|  | 2000 Sub 2 | 0.7156 | 63.88% | 0.6060 | 0.6250 | 0.5882 |
|  | 1500 Sub 3 | 0.8482 | 75.10% | 0.7692 | 0.7894 | 0.7500 |
|  | 1000 Sub 4 | 0.8297 | 80.55% | 0.7991 | 0.7368 | 0.8752 |

**Table S3:** Performance evaluation of various subsets using XGBoost

|  | Feature | AUC | ACC | F1 score | PRE | REC |
| --- | --- | --- | --- | --- | --- | --- |
| Without FS | 5334 | 0.7293 | 65.90% | 0.6667 | 0.6810 | 0.6521 |
| FS Round 1  FS | 5000 Sub 1 | 0.7314 | 65.90% | 0.6667 | 0.6810 | 0.6521 |
|  | 4500 Sub 2 | 0.6487 | 56.81% | 0.6274 | 0.7272 | 0.5517 |
|  | 4000 Sub 3 | 0.7107 | 68.18% | 0.6818 | 0.6818 | 0.6818 |
|  | 3500 Sub 4 | 0.7190 | 65.91% | 0.6341 | 0.591 | 0.684 |
| FS Round 2  mRMR | 2500 Sub 1 | 0.7479 | 70.45% | 0.6976 | 0.6818 | 0.7142 |
|  | 2000 Sub 2 | 0.7334 | 68.18% | 0.6667 | 0.6363 | 0.7000 |
|  | 1500 Sub 3 | 0.7791 | 75.00% | 0.7441 | 0.8000 | 0.6956 |
|  | 1000 Sub 4 | 0.783 | 72.73% | 0.727 | 0.727 | 0.727 |

**Table S4:** Performance evaluation of various subsets using Light GBM

|  | Feature | AUC | ACC | F1 score | PRE | REC |
| --- | --- | --- | --- | --- | --- | --- |
| Without FS | 5334 | 0.7582 | 59.09% | 0.5912 | 0.6111 | 0.6103 |
| FS Round 1  FS | 5000 Sub 1 | 0.8204 | 66.67% | 0.6667 | 0.6667 | 0.6667 |
|  | 4500 Sub 2 | 0.7969 | 75.00% | 0.7500 | 0.7491 | 0.7328 |
|  | 4000 Sub 3 | 0.8469 | 72.22% | 0.7109 | 0.7222 | 0.7222 |
|  | 3500 Sub 4 | 0.7647 | 63.64% | 0.6435 | 0.6515 | 0.6624 |
| FS Round 2  mRMR | 2500 Sub 1 | 0.7906 | 69.44% | 0.6845 | 0.6901 | 0.6887 |
|  | 2000 Sub 2 | 0.8395 | 75.00% | 0.7495 | 0.7520 | 0.7432 |
|  | 1500 Sub 3 | 0.7860 | 75.00% | 0.7271 | 0.7323 | 0.7414 |
|  | 1000 Sub 4 | 0.7548 | 75.86% | 0.7627 | 0.7617 | 0.7612 |

**Table S5:** Performance evaluation of various subsets using CNN

|  | Feature | AUC | ACC | F1 score | PRE | REC |
| --- | --- | --- | --- | --- | --- | --- |
| Without FS | 5334 | 0.7115 | 58.62% | 0.5384 | 0.5384 | 0.5384 |
| FS Round 1  FS | 5000 Sub 1 | 0.6380 | 62.06% | 0.6310 | 0.6222 | 0.6108 |
|  | 4500 Sub 2 | 0.6952 | 55.17% | 0.6976 | 0.6524 | 0.5357 |
|  | 4000 Sub 3 | 0.7000 | 65.51% | 0.7499 | 0.6125 | 0.6291 |
|  | 3500 Sub 4 | 0.7252 | 68.96% | 0.5715 | 0.4613 | 0.7514 |
| FS Round 2  mRMR | 2500 Sub 1 | 0.6380 | 65.51% | 0.7368 | 0.6792 | 0.6086 |
|  | 2000 Sub 2 | 0.6809 | 68.96% | 0.7428 | 0.8667 | 0.6500 |
|  | 1500 Sub 3 | 0.7428 | 75.86% | 0.8011 | 0.9333 | 0.7110 |
|  | 1000 Sub 4 | 0.8343 | 81.81% | 0.8462 | 1.000 | 0.7333 |

1. **Supplementary Figures**


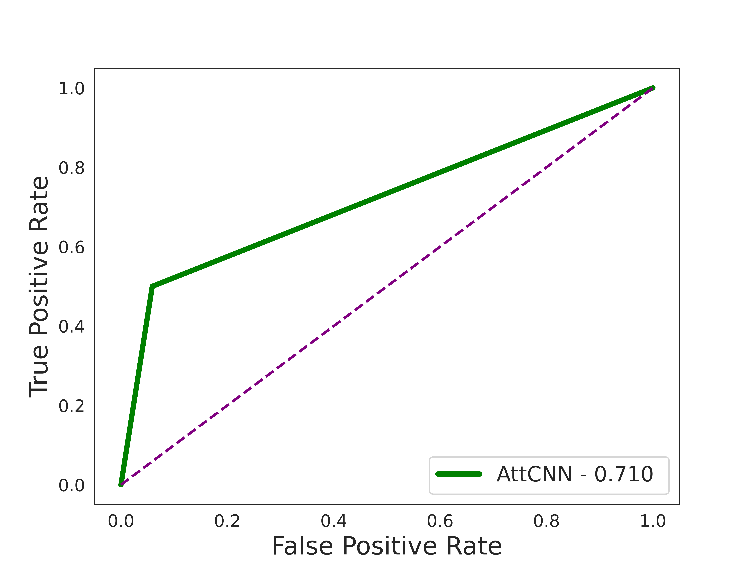

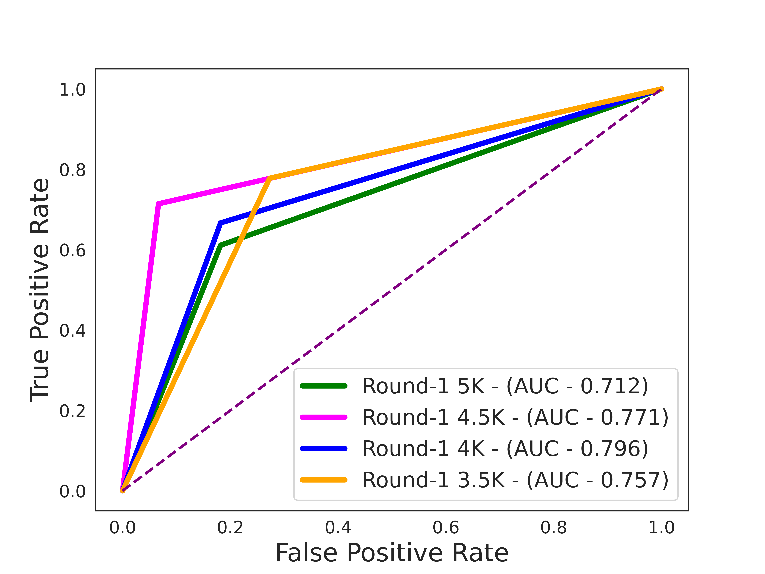


1. (b)


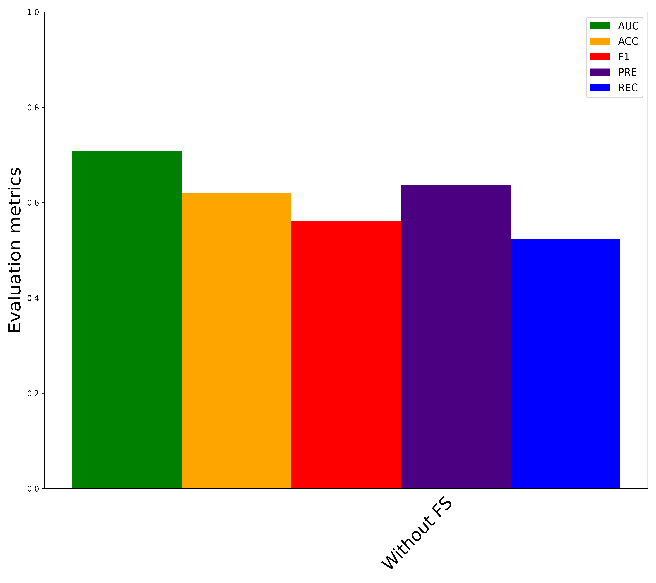

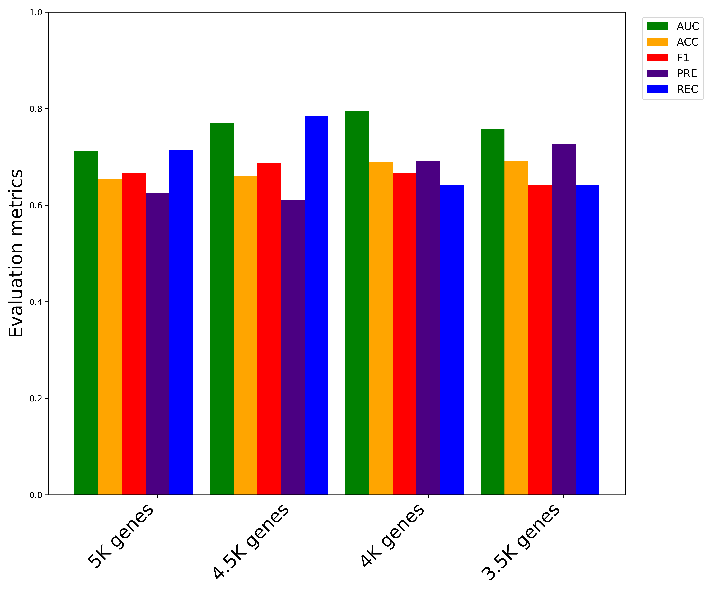


(c) (d)

**Fig. S1:** Evaluation of gene subset classification performance using AttCNN. (a) ROC curve for the initial gene subset (5,334 genes) selected based on p-value thresholds without feature selection. (b) ROC curves for different gene subsets obtained from the first round of feature selection (FS Round 1) using the Fisher score. (c) Performance metrics for the initial gene subset without feature selection. (d) Comparative evaluation of different gene subsets (5K, 4.5K, 4K, 3.5K genes), highlighting the improved classification performance with feature selection, where the 3.5K subset achieved the highest accuracy and AUC in Round 1.


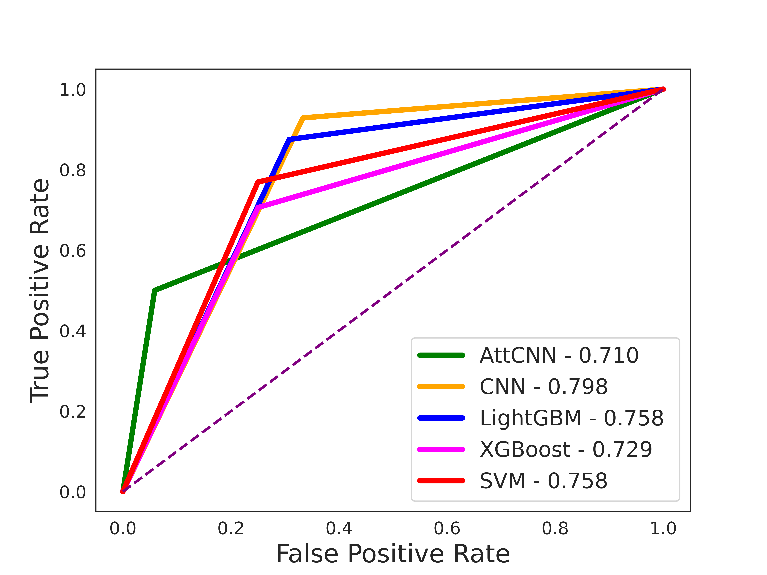

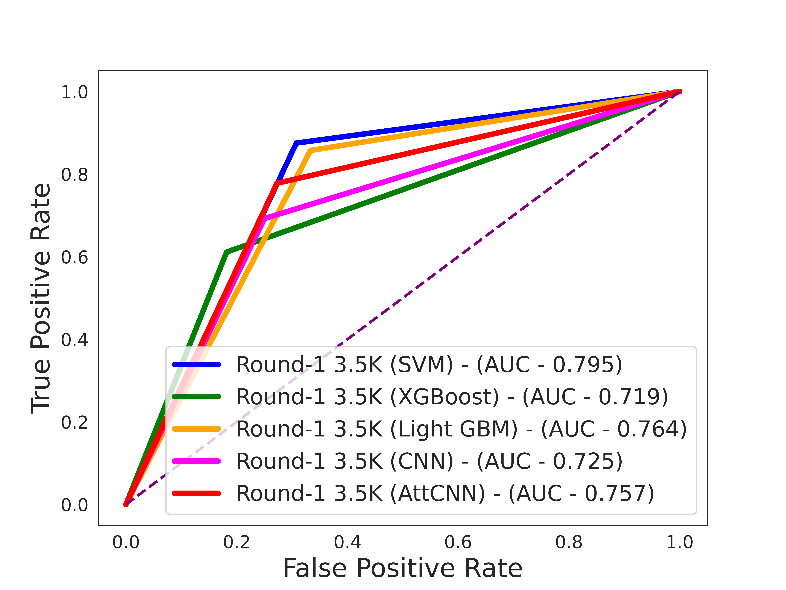


1. (b)


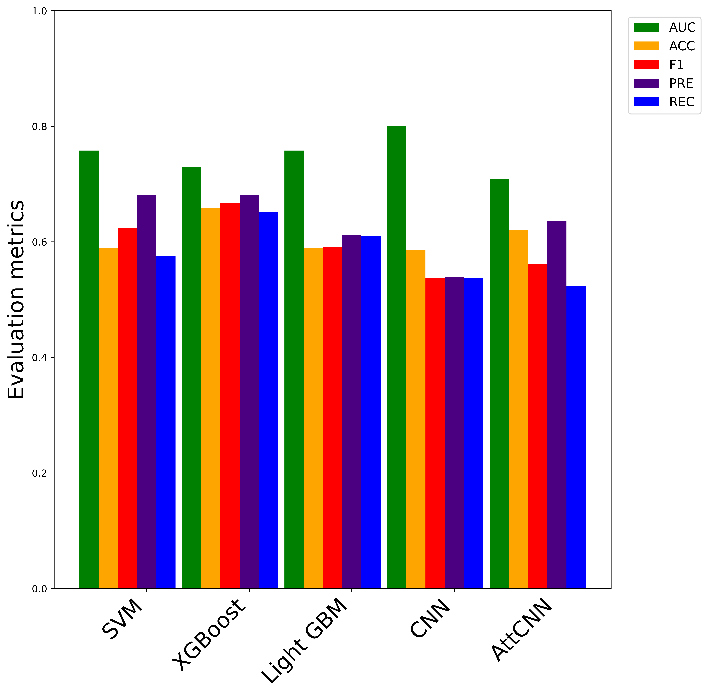

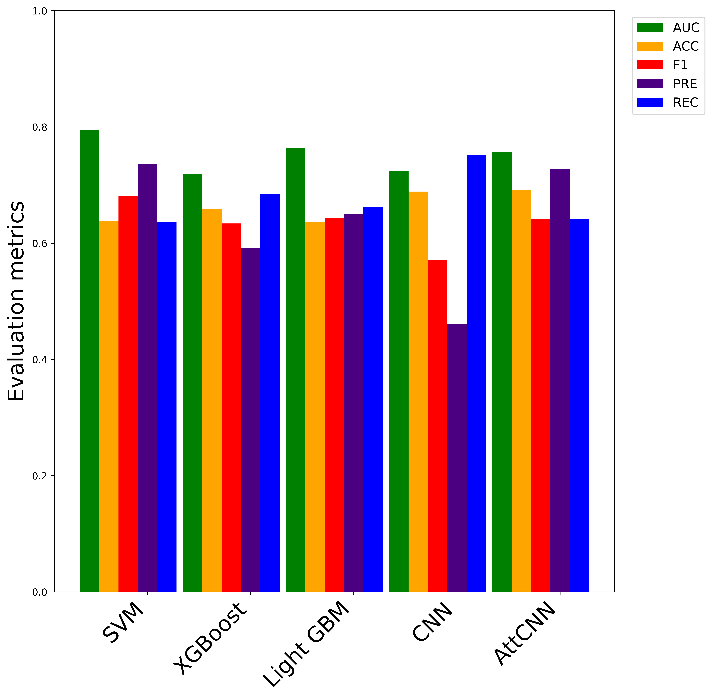


(c) (d)

**Fig. S2:** Evaluation of classification models across feature selection stages. (a) ROC curves illustrating the performance of different classifiers (SVM, XGBoost, LightGBM, CNN, and AttCNN) on the initial gene set (5,334 genes) without feature selection. (b) ROC curves for the 3,500-gene subset obtained from FS Round 1, showing improvements across classifiers. (c) Bar chart comparing classification metrics (AUC, ACC, F1, Precision, Recall) for different models on the initial gene set. (d) Performance comparison of classifiers on the 3,500-gene subset, demonstrating AttCNN’s superior classification capability.


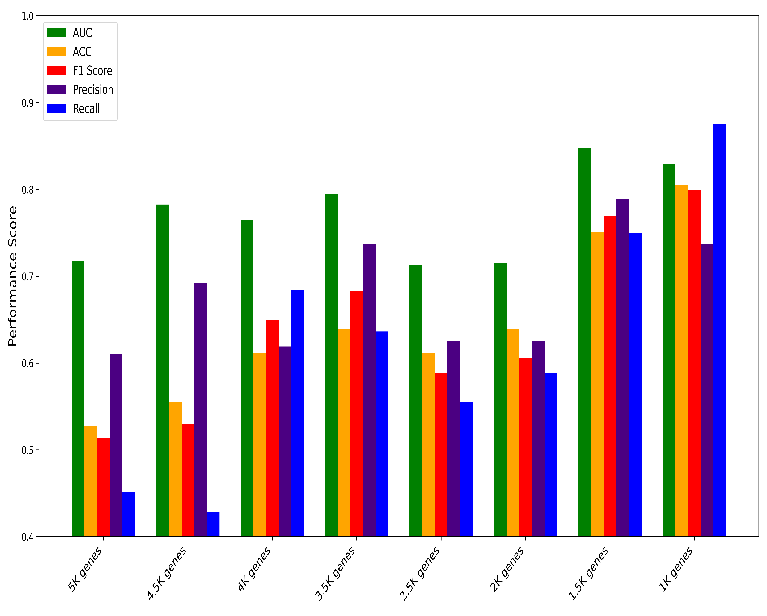

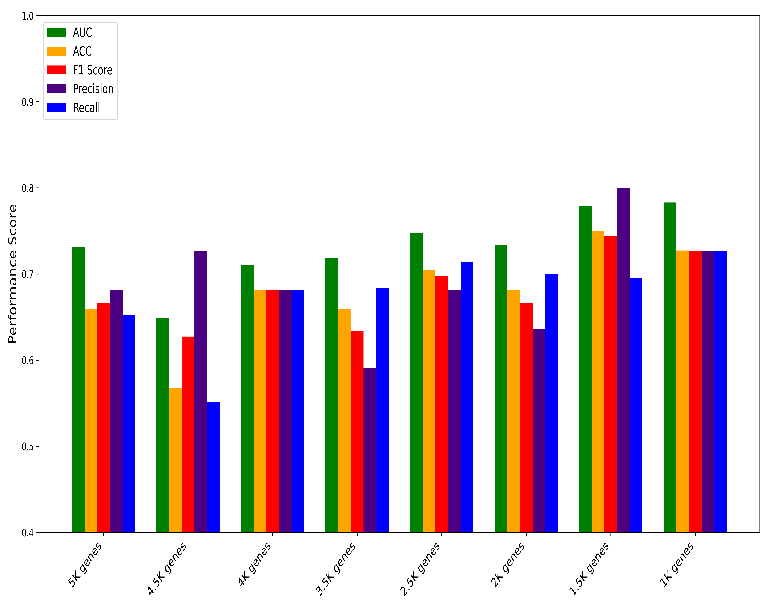


1. (b)


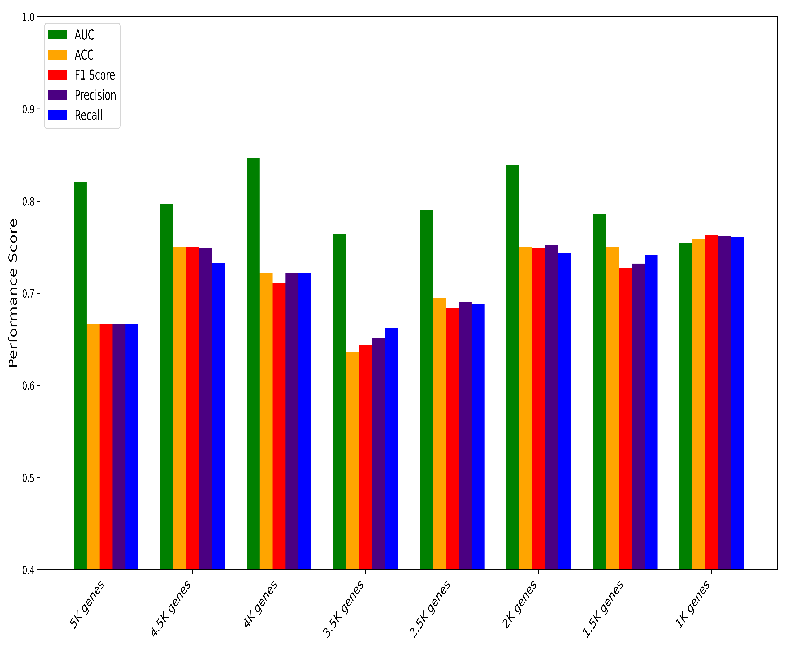

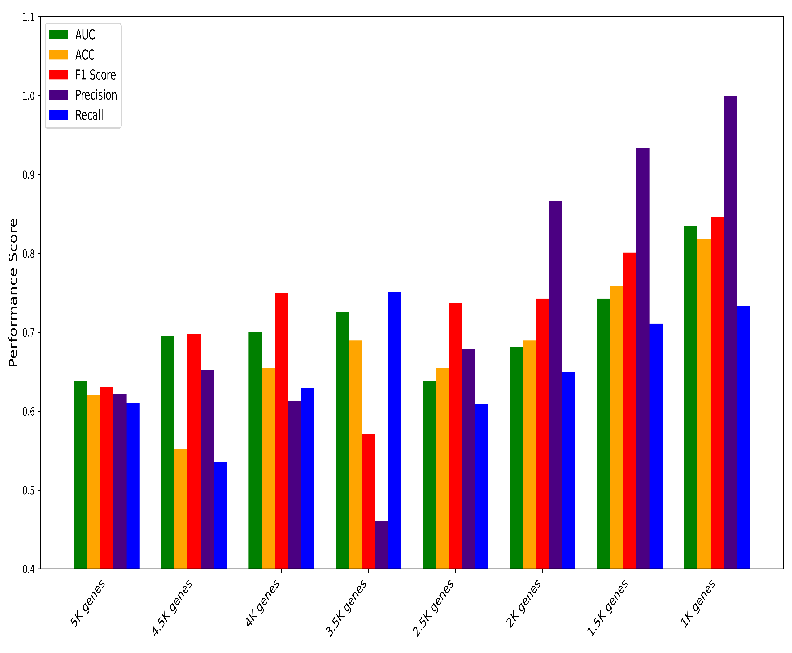


(c) (d)

**Fig. S3:** Performance evaluation of different subsets FS-mRMR across various classifiers. Bar plots depict the performance scores (AUC, ACC, F1 Score, Precision, and Recall) for different gene subsets in (a) SVM, (b) XGBoost, (c) LightGBM, and (d) CNN classifiers.


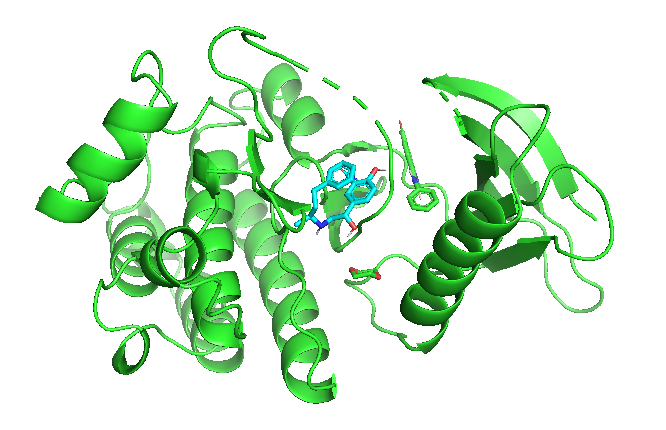

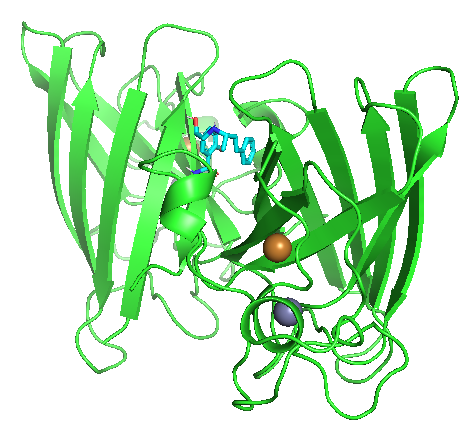


1. (b)


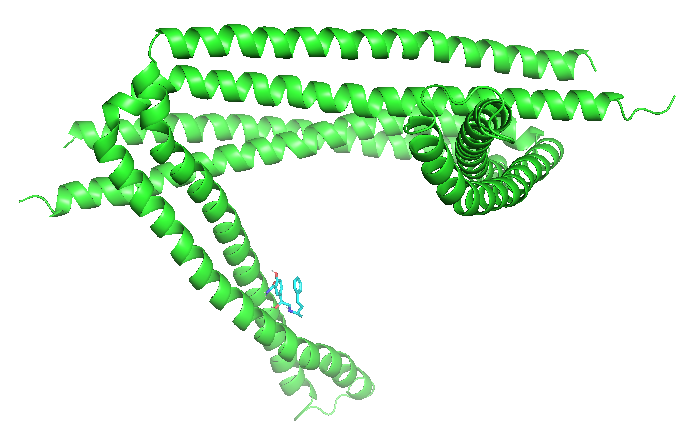


(c)

**Fig. S4:** Molecular docking configurations of the three prominent hub genes with the Labetalol drug compound. (a) HK2, (b) SOD1, (c) SH3BP5. Proteins are depicted in green, while ligands are shown in blue.
